# SULFs in human neoplasia: implication as progression and prognosis factors

**DOI:** 10.1186/1479-5876-9-72

**Published:** 2011-05-21

**Authors:** Caroline Bret, Jérôme Moreaux, Jean-François Schved, Dirk Hose, Bernard Klein

**Affiliations:** 1 INSERM U847, Institut de Recherche en Biothérapie, CHRU de Montpellier, France; 2 Laboratoire Central d'Hématologie, CHRU de Montpellier, France; 3 UFR de Médecine, Université de Montpellier, France; 4 Medizinische Klinik und Poliklinik V, Heidelberg, Germany; 5 Nationales Centrum für Tumorerkrankungen, INF350, Heidelberg, Germany

## Abstract

**Background:**

The sulfation pattern of heparan sulfate chains influences signaling events mediated by heparan sulfate proteoglycans located on cell surface. SULF1 and SULF2 are two endosulfatases able to cleave specific 6-O sulfate groups within the heparan chains. Their action can modulate signaling processes, many of which with key relevance for cancer development and expansion. SULF1 has been associated with tumor suppressor effects in various models of cancer, whereas SULF2 dysregulation was in relation with protumorigenic actions. However, other observations argue for contradictory effects of these sulfatases in cancer, suggesting the complexity of their action in the tumor microenvironment.

**Methods:**

We compared the expression of the genes encoding SULF1, SULF2 and heparan sulfate proteoglycans in a large panel of cancer samples to their normal tissue counterparts using publicly available gene expression data, including the data obtained from two cohorts of newly-diagnosed multiple myeloma patients, the Oncomine Cancer Microarray database, the Amazonia data base and the ITTACA database. We also analysed prognosis data in relation with these databases.

**Results:**

We demonstrated that *SULF2 *expression in primary multiple myeloma cells was associated with a poor prognosis in two independent large cohorts of patients. It remained an independent predictor when considered together with conventional multiple myeloma prognosis factors. Besides, we observed an over-representation of *SULF2 *gene expression in skin cancer, colorectal carcinoma, testicular teratoma and liver cancer compared to their normal tissue counterpart. We found that *SULF2 *was significantly over-expressed in high grade uveal melanoma compared to low grade and in patients presenting colorectal carcinoma compared to benign colon adenoma.

We observed that, in addition to previous observations, *SULF1 *gene expression was increased in T prolymphocytic leukemia, acute myeloid leukemia and in renal carcinoma compared to corresponding normal tissues. Furthermore, we found that high *SULF1 *expression was associated with a poor prognosis in lung adenocarcinoma.

Finally, *SULF1 *and *SULF2 *were simultaneously overexpressed in 6 cancer types: brain, breast, head and neck, renal, skin and testicular cancers.

**Conclusions:**

*SULF1 *and *SULF2 *are overexpressed in various human cancer types and can be associated to progression and prognosis. Targeting SULF1 and/or SULF2 could be interesting strategies to develop novel cancer therapies.

## Background

Heparan sulfate proteoglycans (HSPGs) are negatively-charged proteins located at a high cell density on various cell types or released into the extracellular matrix. As HSPGs bind a large diversity of molecules: growth factors (GF), cytokines, chemokines, morphogens, matrix ligands and cell surface molecules, they are involved in cell signaling as co-receptors [1]. The complexity of the heparan sulfate (HS) chains is based on modifications as epimerisation, de-acetylation and sulfation. These phenomenons strongly influence the ligand binding properties of HSPGs and define the concept of "HS code". The sulfation pattern in glucosamines and uronic acids is dynamically regulated during many cellular processes, generating diversity of the chains and thus diversity of binding. Such mechanisms are regulated by sulfotransferases involved in the biosynthesis of HS. Another class of enzymes is also implicated at the extracellular level: the sulfatases sulfatase 1 (SULF1) and sulfatase 2 (SULF2). Initially cloned in 2002 [2], these secreted enzymes display endoglucosamine 6-sulfatase activity. The expression of the genes encoding these enzymes is developmentally regulated. In murine model, simultaneous disruption of both *SULF1 *and *SULF2 *leads to perinatal lethality and developmental defects underlying overlapping and essential roles during development [3]. However, SULF1-deficient mice did not present any abnormal phenotype whereas SULF2-knock-out mice displayed a small but significant reduction in litter size and body weight, and a hydrocephalus at birth resulting in a life span shorter than 2 weeks [4].

Owing to the involvement of HSPGs as coreceptors of cell communication molecules, the role of these HSPG modifying enzymes in human tumorigenesis is actively investigated. Despite similar substrate specificity, SULF1 has mainly tumor suppressor functions whereas SULF2 presents tumor promoting functions. In this article, we focused on recent and challenging data describing the implication of SULF1 and SULF2 in human neoplasia.

## Methods

### Databases

*SULF1 *and *SULF2 *gene expression levels in normal or malignant human tissues or cell lines were obtained from the Oncomine Cancer Microarray database (http://www.oncomine.org) [5], the Amazonia database (http://amazonia.montp.inserm.fr/) [6] and the ITTACA database (Integrated Tumor Transcriptome Array and Clinical data Analysis) developed by the Institute Curie Bioinformatics group and the Institute Curie, CNRS UMR144 (http://bioinfo-out.curie.fr/ittaca/) [7]. Gene expression data only obtained from a single study using the same methodology were compared. All data were log transformed, median centered per array and the standard deviation was normalized to one per array.

### Primary myeloma cells

Multiple Myeloma cells (MMC) were purified from 206 patients with newly-diagnosed MM after written informed consent was given at the University hospitals of Heidelberg (Germany) or Montpellier (France). The study was approved by the ethics boards of Heidelberg University and Montpellier University. After Ficoll-density gradient centrifugation, plasma cells were purified using anti-CD138 MACS microbeads (Miltenyi Biotech, Bergisch Gladbach, Germany). Microarray experiments were performed in DNA microarray platform of the Institute of Research in Biotherapy at the Montpellier University Hospital (France) http://irb.montp.inserm.fr/en/index.php?page=Plateau&IdEquipe=6. The .CEL files and MAS5 files have been deposited in the ArrayExpress public database, under accession number E-MTAB-362. We also used Affymetrix data of a cohort of 345 purified MMC from previously untreated patients from the Arkansas Cancer Research Center (ACRC, Little Rock, AR). These data are publicly available *via *the online Gene Expression Omnibus (Gene Expression Profile of Multiple Myeloma, accession number GSE2658, http://www.ncbi.nlm.nih.gov/geo/).

### Statistical analysis

Statistical comparisons were done with Student t-tests. The event free or overall survival of subgroups of patients was compared with the log-rank test and survival curves computed with the Kaplan-Meier method. The prognostic values of parameters were compared with univariate or multivariate Cox analysis. Statistical tests were performed with the software package SPSS 12.0 (SPSS, Chicago, IL).

## Results and discussion

### Tumor suppressor functions of SULF1

Expression of *SULF1 *mRNA can be detected in several normal human tissues, as observed by Morimoto-Tomita et al. [2] in a panel of 24 tissue types, the highest levels being found in testes, stomach, skeletal muscle, lung, and kidney. *SULF1 *down-regulation has been described in human primary tumorous samples and/or cell lines in ovarian cancer [8-10], hepatocellular carcinoma [11], breast cancer [12], gastric cancer [12], kidney cancer [12], prostatic stromal cells from benign prostatic hyperplasia samples [13] and head and neck squamous cell carcinoma (SCCHN) cell lines [14]. This low expression level is mostly explained by epigenetic silencing mediated by hypermethylation of the promoter of the gene encoding SULF1 [9,12].

Considering that HSPG sulfation pattern drives in part cell communication molecule binding [15-17], a loss of *SULF1 *expression is expected to disrupt the effects of these cell communication molecules during malignancies. It has been observed that this down-regulation results in increased sulfation of HS chains and could produce the stabilization of ternary receptor complexes, leading to an increased in GF signalling, as described for heparin-binding epidermal growth factor-like growth factor (HB-EGF), fibroblast growth factor 2 (FGF2) or amphiregulin in ovarian cancer [8], SCCHN cell lines [14], hepatocellular carcinoma [18] or in breast cancer [19]. This modulation of GF effects can affect major events including proliferation of cancer cells. A forced expression of *SULF1 *induced growth inhibition of neck squamous cell carcinoma cell lines *in vitro*[14]. A marked reduction of the growth of myeloma or breast cancer cell lines was observed in severe combined immunodeficient (SCID) mice when injected cell lines were transfected with *SULF1 *cDNA [20,21]. Forced expression of *SULF1 *also significantly delayed the growth of hepatocellular carcinoma cell lines xenografts in nude mice [22].

These different models also argued the role of SULF1 as an inhibitor of motility, invasion and angiogenesis and as a protein linked to drug-induced apoptosis. Hepatocyte growth factor (HGF)-mediated motility and invasion were attenuated in SCCHN cell lines displaying an overexpression of this sulfatase [14]. Xenografts derived from *SULF1*-expressing carcinoma cells presented a significantly reduced ability of vascular HS to promote a stable complex between FGF2 and its specific receptor with an inhibition of angiogenesis as a result. The down-regulation of *SULF1 *in human umbilical vein endothelial cells (HUVECs) could increase vascular endothelial growth factor (VEGF)-induced angiogenic response [21]. In hepatocellular carcinoma (HCC), SULF1 enhanced the induction of apoptosis by the histone deacetylase (HDAC) inhibitors *in vitro*[22]. The doxorubicin and apicidin-induced apoptosis was significantly increased of in HCC cell lines expressing *SULF1*. In addition, the anti-tumor effects of these drugs were enhanced *in vivo *when a xenograft was established from *SULF1*-expressing HCC [23]. SCCHN-transfected cell lines displayed significant growth inhibition concomitant with an increased sensitivity to staurosporine- and cisplatin-induced apoptosis [14].

Altogether, these data suggest that the widespread *SULF1 *down-regulation in cancer might be an important contributor to the carcinogenesis process.

### SULF2, a protumorigenic endosulfatase

**Figure 1:**
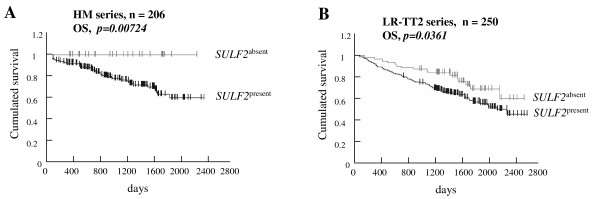
**Overall survival (OS) related to *SULF2 *gene expression in two independent multiple myeloma patient series**. Data are Kaplan-Meier curves of patients displaying an "absent call" *versus *patients displaying a "present call". A. Cohort of 206 patients (HM) from Montpellier (France) and Heidelberg (Germany). B. Cohort of 250 patients (LR-TT2) of Little-Rock.

**Figure 2:**
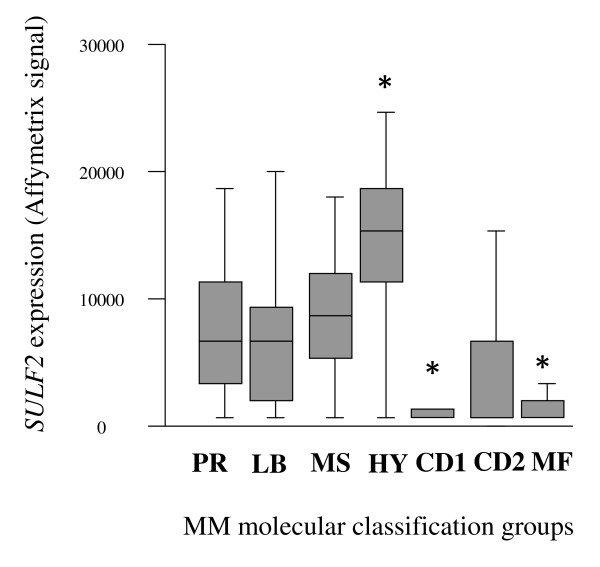
***SULF2 *expression in the 7 groups of the molecular classification of multiple myeloma**. The expression of *SULF2 *in LR-TT2 cohort was investigated in the 7 groups of the molecular classification of multiple myeloma. PR: proliferation, LB: low bone disease, MS: MMSET, HY: hyperdiploid, CD1: Cyclin D1, CD2: Cyclin D2, MF: MAF.

The implication of SULF2 in cancer was less studied than that of SULF1. However, most of the studies documented a protumorigenic role of SULF2 at the opposite of that of SULF1. Lemjabbar-Alaoui et al. [24] observed an induction of *SULF2 *expression in human lung adenocarcinoma and squamous cell carcinoma with a mean increase of 3-fold compared to normal lung. They could obtain a loss of the transformed phenotype of lung carcinoma cell lines when silencing *SULF2 *expression with short-hairpin RNA (sh-RNA). The knock-out of *SULF2 *in these cell lines also resulted in a decreased tumor formation when grafted to nude mice. Besides, SULF2 was shown to modulate the bioavailability of wingless-type MMTV integration site family (Wnt) ligands, a critical canonical cascade reactivated in several tumors [25]. An up-regulation of *SULF2 *mRNA was also observed in human or murine breast cancers compared to normal breast tissues [26]. *SULF2 *was up-regulated in primary HCC samples, as well as in HCC cell lines [11]. It resulted in an activation of mitogen-activated protein kinase (MAPK) and v-akt murine thymoma viral oncogene homolog 1 (Akt) pathways with an increased cell growth *in vitro *and *in vivo*. In multiple myeloma (MM), we had previoulsy reported an overexpression of *SULF2 *gene in primary myeloma cells of newly-diagnosed myeloma compared to normal bone marrow plasma cells [27]. In this study, we demonstrate for the first time that *SULF2 *expression in primary multiple myeloma cells (MMCs) ("absent" *versus *"present" Affymetrix call) was associated with a poor prognosis in two independent large cohorts of myeloma patients at diagnosis (206 patients in the cohort of Heidelberg-Montpellier and 250 patients in the cohort of Little-Rock previously described [28], Figure 1A and 1B). Patients with *SULF2*^*absent *^MMCs had a significant increased overall survival compared with patients with *SULF2*^*present *^MMCs (*p *= 0.007 in the Heidelberg-Montpellier cohort and *p *= 0.03 in the Little-Rock cohort), after high-dose therapy and stem cell transplantation. In a Cox proportional hazard model (Table 1), the absence or the presence of *SULF2 *(*p = 0.007*, hazard ratio = 4.08) and ISS stage (*p = 0.001*, hazard ratio = 1.73) were independently predictive for overall survival (*p = 0.02 *and *p = 0.001*, respectively). If *SULF2 *expression was tested together with classical prognostic factors, i.e., serum albumin and serum beta 2 microglobulin (b2M), *SULF2 *expression (*p = 0.03*) and b2M (*p = 0.0001*) remained independent prognostic factors. *SULF2 *expression was an independent prognostic factor of spiked MMSET expression, that is an indicator of t(4;14) translocation [29] (*p = 0.023 *and *p = 0.028 *respectively), of the myeloma high risk score (HRS) [30] (*p = 0.01 *and *p = 0.002 *respectively), of the growth proliferation index [31] (*p = 0.01 *and *p = 0.0001 *respectively), of the IFM score [32] (*p = 0.01 *and *p = 0.0001 *respectively) and of CD200 expression [33] (*p = 0.02 *and *p = 0.05 *respectively). Investigating the *SULF2 *expression in the 7 groups of the molecular classification [34] of MM, *SULF2 *was significantly overexpressed in the hyperdiploid group and significantly underexpressed in the groups of patients characterized by Cyclin D1 or MAF translocations (Figure 2). We analyzed the correlation between *SULF1 *or *SULF2 *expression and HS proteoglycans expression in our cohort of myeloma patients (syndecan 1-4, glypican 1-6, CD44 isoforms containing the alternatively spliced exon v3, agrin, betaglycan, perlecan, serglycin and testican 1-3)[27]. No significant correlation was found between the expression of the SULFs and of their potential HS proteoglycan targets in MM. When we analyzed the correlation between the expression of the sulfatases and of the genes encoding the transporters and the enzymes involved in HS and chondroïtine sulfate biosynthesis pathway [27], we did not found any correlation for *SULF2 *but we observed a correlation between *SULF1 *expression and *GALK1 *(galactokinase 1) and *SLC2A9 *(solute carrier family 2, facilitated glucose transporter member 9) expression.

**Table 1:** Univariate and multivariate proportional hazards analyses linking *SULF2 *expression to prognosis in HM cohort

HM cohort (OS)			
	**Pronostic variable**	**Proportional hazard ratio**	**P-value**

Univariate Cox analysis	SULF2 ISS	4.08 1.73	***0.007*** ***0.001***

Multivariate Cox analysis	SULF2 ISS	3.65 1.70	***0.028*** ***0.001***

Univariate Cox analysis	SULF2 b2M Alb	4.08 1.10 1.60	***0.007*** ***0.0001*** ***0.04***

Multivariate Cox analysis	SULF2 b2M Alb	3.49 1.10 1.35	***0.03*** ***0.0001*** ***0.24***

Univariate Cox analysis	SULF2 HRS	4.08 2.30	***0.007*** ***0.002***

Multivariate Cox analysis	SULF2 HRS	4.11 2.31	***0.01*** ***0.002***

Univariate Cox analysis	SULF2 MS group	4.08 2.14	***0.007*** ***0.001***

Multivariate Cox analysis	SULF2 MS group	3.84 1.97	***0.023*** ***0.028***

Univariate Cox analysis	SULF2 IFM score	4.08 3.09	***0.007*** ***0.0001***

Multivariate Cox analysis	SULF2 IFM score	4.29 3.22	***0.014*** ***0.0001***

Univariate Cox analysis	SULF2 GPI	4.08 2.21	***0.007*** ***0.0001***

Multivariate Cox analysis	SULF2 GPI	4.47 2.25	***0.011*** ***0.0001***

Univariate Cox analysis	SULF2 MYEOV	4.08 3.16	***0.007*** ***0.05***

Multivariate Cox analysis	SULF2 MYEOV	3.71 2.76	***0.026*** ***0.08***

Univariate Cox analysis	SULF2 CD200	4.08 2.05	***0.007*** ***0.03***

Multivariate Cox analysis	SULF2 CD200	3.86 1.03	***0.02*** ***0.05***

Univariate analyses were done to screen for prognostic variables linked to *SULF2 *expression using Cox proportional hazards regression. The Cox model was also used for multivariate analysis to identify the most significant variables related to survival (OS): ISS (international staging system), b2M (beta-2 microglobulin), Alb (Albumin), HRS (High Risk Score), MS group (MMSET group), IFM score (IFM score), GPI (Growth Proliferation Index), MYEOV and CD200. P-values are in bold and italic when a significant result was obtained (p < 0.05).

In HCC model, sh-RNA targeting *SULF2 *induced an inhibition of HCC cell lines proliferation and migration *in vitro*. In nude mice, SULF2 could significantly promote HCC xenograft growth. Besides, forced expression of this enzyme increased glypican-3 expression level, this membrane-anchored HSPG being involved in Wnt pathway, FGF signaling and cell proliferation [35]. Moreover, in patients with HCC, high levels of SULF2 were associated with a worse prognosis [11]. In human pancreatic carcinoma, the SULFs are up-regulated and it has been observed that the silencing of *SULF2 *could lead to an inhibition of Wnt signalling and of cell growth [36]. In order to explore the pathogenesis of glioblastoma, Johansson et al. generated a mouse glioma model using a recombinant Moloney murine leukemia virus encoding the platelet-derived growth factor B-chain and intra-cerebrally injected in newborn mice [37]. Using expression profiling, they identified markers of gliomagenesis, *SULF2 *appearing among the candidate cancer-causing genes.

In addition to its contribution during tumor growth development, SULF2 could be implicated in resistance to cancer treatment, as recently suggested by Moussay et al. [38]. A comparison of gene expression profiles of sensitive and resistant clones of chronic lymphocytic leukemia obtained from patients treated by fludarabine was performed. Together with *v-myc myelocytomatosis viral oncogene homolog *(*MYC*), *SULF2 *transcripts were significantly over-represented in cells of patients resistant to fludarabine.

**Figure 3:**
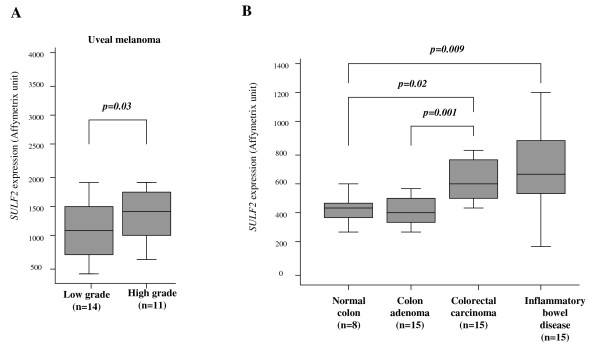
**Association between *SULF2 *expression and progression in various cancers**. A. *SULF2 *gene expression in uveal melanoma [55]. B. *SULF2 *gene expression in samples of normal colon, adenoma, colorectal carcinoma and inflammatory bowel disease [41]. *P *values are indicated in each panel.

Recently, *SULF2 *gene expression was investigated in a large panel of cancer samples, using the ONCOMINE microarray database (https://www.oncomine.org 4.3 research edition) [39]. Rosen et al. demonstrated an overexpression of *SULF2 *in several cancers including brain, breast, tongue and renal carcinomas [39]. In addition to these observations, we found that other cancer types displayed an over-representation of *SULF2 *gene expression compared to their tissue counterpart: skin (*p = 2.26E-4 *and *p = 1E-3 *[40]), colorectal carcinoma (*p = 0.02 *[41]), testicular teratoma (*p = 6E-3 *[42]) and liver cancer (*p = 1.9E-4 *and *p = 2E-3 *[43]). Using the ITTACA database (Integrated Tumor Transcriptome Array and Clinical data Analysis, http://bioinfo-out.curie.fr/ittaca/)[7] and the AMAZONIA database [6], we searched to identify if *SULF2 *expression could be associated with tumor progression in these cancer types. Interestingly, we found that *SULF2 *was significantly over-expressed in high grade uveal melanoma compared to low grade (*p *= 0.03, Figure 3A). Furthermore, *SULF2 *was also overexpressed in patients presenting colorectal carcinoma compared to benign colon adenoma (*p *= 0.001, Figure 3B).

These different data lend support for a protumorigenic effect of *SULF2 *overexpressed by many tumor cell types.

### Challenging observations concerning SULF1 and SULF2 in cancer

**Figure 4:**
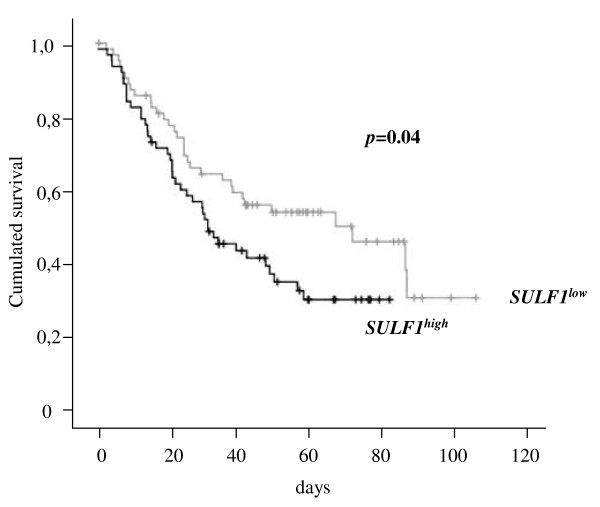
**Overall survival (OS) related to *SULF1 *gene expression in a lung adenocarcinoma patient cohort**. Data are Kaplan-Meier curves of patients displaying a low *SULF1 *expression (n = 64) *versus *patients displaying a high *SULF1 *expression (n = 63, median cutoff) [47].

Using the ONCOMINE microarray database, Rosen et al. shown that, in contrast to the down-regulation of *SULF1 *reported in various tumor models, *SULF1 *gene expression was increased in a large range of cancers compared to their corresponding normal tissues [39]. *SULF1 *was clearly over-expressed in adrenal carcinoma, brain cancer, breast carcinoma, colon adenocarcinoma, skin carcinoma, esophageal and gastric cancers, head and neck cancers, lung cancer, mesothelioma, pancreatic cancer, sarcoma and germ line/testicular cancer [39]. In addition, we found that other cancer types displayed an over-representation of *SULF1 *gene expression: T prolymphocytic leukemia (*p = 0.01 *[44]), acute myeloid leukemia (*p = 0.004 *[45]) and renal carcinoma (*p < 0.001 *[46]). These data challenge the above concept of SULF1 as a tumor suppressor effector. Using the ITTACA database, we aimed to identify if *SULF1 *expression could be associated with tumor progression or bad prognosis in cancers. Indeed, we found that high *SULF1 *expression was associated with a poor prognosis in lung adenocarcinoma (Figure 4) [47]. Although *SULF1 *was overexpressed in breast cancer compared to its normal counterpart [39,48,49], we did not found any significant association between *SULF1 *expression and survival in breast cancer using data from two independent studies (data not shown).

Some studies have brought some explanations about this contradictory contribution to carcinogenesis. In pancreatic cancer cells, the expression of *SULF1 *in xenograft models was associated with a markedly reduced growth potential, but with an increase in the basal invasiveness of these cells [50]. Recently, Sahota and Dhoot [51] demonstrated in quail model the possibility of alternative splicing of *SULF1 *gene, generating a novel shorter isoform called *SULF1B*. While the previously described SULF1 (SULF1A) enhanced Wnt signaling, SULF1B inhibited Wnt signaling and promoted angiogenesis. Such splicing has not been yet described in human tissues but could be of interest, in particular in cancer development. In mutiple myeloma, we previously observed an overexpression of SULF1 by bone marrow stromal cells, whereas primary malignant plasma cells did not express the gene encoding for this sulfatase. Besides, *SULF1 *was expressed by some human myeloma cell lines (HMCLs), emphasizing that these HMCLs can express environment genes, making it possible to escape from environment dependence [27].

Whereas SULF2 is considered as being associated with protumorigenic effects, as reviewed above, a few challenging studies argue for a tumor suppressor effect of this protein. In contrast with our report that *SULF2 *expression in primary malignant plasma cells is associated with poor overall survival [27], Dai et al. [20] observed that a forced expression of SULF2 reduced the growth of myeloma cell lines in SCID mice. Thus, they concluded to a similar action of SULF1 and SULF2 on myeloma cells expansion through the modification of HS sulfation pattern and its consequence in medullar microenvironment.

In addition to this *in vivo *observation, two studies demonstrated that SULF2 is induced by p53 tumor suppressor. Adamsen et al. [52] firstly suggested that *SULF2 *was a putative p53 target gene in colon cancer cells treated by 5-fluorouracil. Inducible p53 knockdown cell lines of multiple cancer types were generated by Chau et al. [53] and their gene expression profiles were compared to the initial cell lines. This method led to the identification of downstream targets of p53. *SULF2 *was found to be a direct transcriptional target of p53 that could bind to the *SULF2 *promoter, in particular in the context of DNA-damaged-induced senescence, in accordance with the observation of Adamsen.

Interestingly, *SULF1 *was overexpressed in 6/7 cancer types characterized by *SULF2 *overexpression compared to normal tissue counterparts (Table 2). Several HS proteoglycans have been identified so far - syndecan 1-4, glypican 1-6, CD44 isoforms containing the alternatively spliced exon v3, agrin, betaglycan, perlecan, serglycin and testican 1-3 - and their gene expression could be evaluated by microarrays [27]. In cancer samples displaying an overexpression of *SULF1 *and/or *SULF2 *compared to their normal counterparts, we systematically observed on overexpression of at least one HS proteoglycans (Table 2). The functional consequences of the presence of the two forms of extracellular sulfatases in human cancer have not been described and could be of interest.

**Table 2:** Expression of genes encoding *SULF1, SULF2 *and heparan sulfate proteoglycans in human cancer samples in comparison with their normal counterpart

		Gene overexpressed in cancer samples in comparison to their normal tissue counterpart									
		
Cancer sample type	Datasets	SULF1	SULF2	Syndecan 1-4	Glypican 1-6	CD44 isoforms containing the alternatively spliced exon v3	Agrin	Betaglycan	Perlecan	Serglycin	Testican 1-3
Leukemia	33	**Yes**	No	No	No	No	No	No	**Yes**	**Yes**	**Yes**

Adrenal cancer	2	**Yes**	No	No	No	No	No	No	No	No	No

Brain cancer	23	**Yes**	**Yes**	**Yes**	**Yes**	**Yes**	**Yes**	**Yes**	**Yes**	**Yes**	No

Breast cancer	44	**Yes**	**Yes**	**Yes**	No	**Yes**	No	No	No	No	**Yes**

Colon cancer	12	**Yes**	No	No	No	**Yes**	No	No	No	No	No

Esophageal cancer	4	**Yes**	No	**Yes**	**Yes**	**Yes**	**Yes**	**Yes**	**Yes**	**Yes**	No

Gastric cancer	5	**Yes**	No	No	No	No	No	No	**Yes**	No	**Yes**

Head & Neck cancer	5	**Yes**	**Yes**	**Yes**	**Yes**	**Yes**	No	No	**Yes**	**Yes**	No

Liver cancer	4	No	**Yes**	No	No	No	No	No	No	No	No

Lung cancer	16	**Yes**	No	No	No	No	**Yes**	No	No	No	**Yes**

Mesothelioma	3	**Yes**	No	No	No	No	No	No	No	No	No

Pancreatic cancer	6	**Yes**	No	**Yes**	No	No	No	No	**Yes**	**Yes**	**Yes**

Renal	11	**Yes**	**Yes**	No	No	**Yes**	**Yes**	No	**Yes**	No	No

Sarcoma	11	**Yes**	No	No	No	No	No	No	No	No	No

Skin cancer	1	**Yes**	**Yes**	No	No	No	No	No	No	No	No

Testicular cancer	1	**Yes**	**Yes**	**Yes**	**Yes**	No	**Yes**	No	No	**Yes**	No

Expression data were obtained from the Oncomine Cancer Microarray database. Genes which were overexpressed in cancer cell samples in comparison with their normal counterpart are indicated in this table.

## Conclusions

The secretion of SULF1 and SULF2 raises the possibility for cancer cells to remodel the extra-cellular matrix in their environment, thereby affecting their development and/or the neighbouring host cells. A strong parallelism can be proposed with heparanase, an enzyme able to cleave HS chains, generating bioactive fragments and leading to protumorigenic effects in various models of cancer and metastatic processes [54]. However, if heparanase is clearly associated to protumorigenic effects, contradictory observations have been made concerning SULF1 and SULF2 contribution in human neoplasia, as we have discussed in this article. These differences could be explained by the various components of tumour microenvironment that can be targeted by SULF1 and SULF2. In addition, most of studies have explored the expression of these sulfatases by cancer cells but, as secreted enzymes, their production by other cell types in cancer stroma could have major effects on signaling mediated by HSPGs. Besides, the possibility of splicing variants could partially explain the different consequences of the surexpression of these proteins in neoplasia. Finally, targeting SULF1 and/or SULF2 could be interesting strategies to develop novel cancer therapies.

## List of abbreviations used

Akt: v-akt murine thymoma viral oncogene homolog 1; b2M: beta 2 microglobulin; FGF: fibroblast growth factor; GF: growth factor; GPI: growth proliferation index; HB-EGF: heparin-binding epidermal growth factor-like growth factor; HCC: hepatocellular carcinoma; HDAC: histone deacetylase; HGF: hepatocyte growth factor; HMCL: human myeloma cell line; HRS: high risk score; HS: heparan sulphate; HSPG: heparan sulfate protéoglycane; HUVEC: human umbilical vein endothelial cells; MAPK: mitogen-activated protein kinase; MM: multiple myeloma; MS: MMSET group; MYC: v-myc myelocytomatosis viral oncogene homolog; OS: overall survival; SCCHN: head and neck squamous cell carcinoma; SCID: severe combined immunodéficiente; sh-RNA: short-hairpin RNA; SULF1: sulfatase 1; SULF2: sulfatase 2; VEGF: vascular endothelial growth factor; Wnt: wingless-type MMTV integration site family.

## Competing interests

The authors declare that they have no competing interests.

## Authors' contributions

CB designed the study, supported data analysis and wrote the paper.

JM was involved in the study design and supported data analysis.

JFS and DH participated in the design of the study.

BK is the senior investigator who designed research and wrote the paper.

All authors read and approved the final manuscript.
